# Computational Insights into the Radical Scavenging Activity and Xanthine Oxidase Inhibition of the Five Anthocyanins Derived from Grape Skin

**DOI:** 10.3390/antiox13091117

**Published:** 2024-09-15

**Authors:** Xiao-Qin Lu, Jindong Li, Bin Wang, Shu Qin

**Affiliations:** Shanxi Center for Testing of Functional Agro-Products, Shanxi Agricultural University, Taiyuan 030031, China; lijindong@sxau.edu.cn (J.L.); wangbin_jczx@163.com (B.W.); qinshu55@126.com (S.Q.)

**Keywords:** anthocyanins, radical scavenging activity, DFT, xanthine oxidase inhibition, molecular docking

## Abstract

Anthocyanins, typical polyphenol compounds in grape skin, have attracted increasing interest due to their health-promoting properties. In this body of work, five representative anthocyanins (Cy-3-*O*-glc, Dp-3-*O*-glc, Pn-3-*O*-glc, Mv-3-*O*-glc, and Pt-3-*O*-glc) were studied using the density functional theory (DFT) to elucidate structure–radical scavenging activity in the relationship and the reaction path underlying the radical-trapping process. Based on thermodynamic parameters involved in HAT, SET-PT, and SPLET mechanisms, along with the structural attributes, it was found that the C4′ hydroxyl group mainly contributes to the radical scavenging activities of the investigated compounds. Pt-3-*O*-glc exhibits a good antioxidant capacity among the five compounds. The preferred radical scavenging mechanisms vary in different phases. For the Pt-3-*O*-glc compound, the calculations indicate the thermodynamically favoured product is benzodioxole, rather than *o*-quinone, displaying considerably reduced energy in double HAT mechanisms. Additionally, the thermodynamic and kinetic calculations indicate that the reaction of ^•^OH into the 4′-OH site of Pt-3-*O*-glc has a lower energy barrier (7.6 kcal/mol), a higher rate constant (5.72 × 10^9^ M^−1^ s^−1^), and exhibits potent ^•^OH radical scavenging properties. Molecular docking results have shown the strong affinity of the studied anthocyanins with the pro-oxidant enzyme xanthine oxidase, displaying their significant role in inhibiting ROS formation.

## 1. Introduction

Reactive oxygen species (ROS) are continuously being produced in living organisms and participate in a range of relevant physiological processes that maintain homeostasis. However, their overproduction would trigger oxidative stress and cause cell damage, resulting in the onset of various diseases, including neurological diseases, cardiovascular disorders, ageing, and cancer [1,2,3,4,5]. Hence, these antioxidants are vital in protecting against ROS therapeutically. In addition, the activation of pro-oxidant enzymes, such as xanthine oxidase (XO), within the peroxisomes is related to the intracellular overproduction of ROS [6]. Xanthine oxidase (XO) can catalyse hypoxanthine in order to produce xanthine, which is then converted to uric acid and concomitantly generates ROS [7]. To overcome the burden of the body’s uric acid and ROS levels, inhibiting key enzyme XO is an effective approach. Thus, searching for efficient antioxidant compounds and potent inhibitors of xanthine oxidase would be an essential strategy for defending against oxidative stress.

Antioxidants from natural products have received tremendous scientific interest as beneficial and health-promoting agents in the past few decades, due to their excellent antioxidant capacity and nontoxic effects on humans [8,9]. Anthocyanins are water-soluble flavonoid pigments abundantly found in plants [10]. Numerous modern technologies have been utilised in experiments to extract and purify anthocyanins from grape skin, which are a widespread by-product of the wine industry and contain anthocyanins, alongside other bioactive compounds [11,12,13]. Meanwhile, a number of studies have suggested that anthocyanins possess various beneficial health effects, including high antioxidant capacity, anti-inflammatory effects, heart protection, and cancer prevention [14,15,16]. All of these excellent characteristics have stimulated a growing interest in the study of the structures, the chemical properties, and the mechanisms involving anthocyanin molecules.

**Figure 1:**
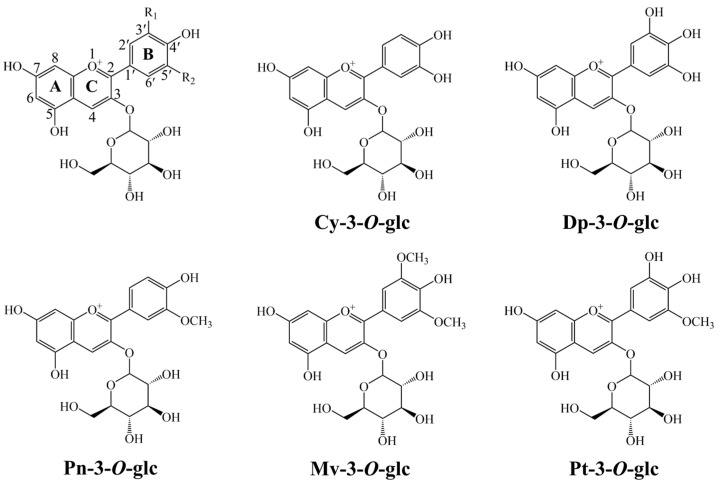
Basis structure and atom numbering sites of anthocyanin and chemical structures of five anthocyanins derived from grape skin.

Anthocyanins are glycosylated analogues of anthocyanidins, which are structurally characterised by the benzopyrylium skeleton. They all hold the basic 2-phenylbenzopyryalium cationic structure at a low pH [17]. However, differences in either the number or position of hydroxyl and/or methoxy groups, as well as the structure and position of sugars attached to the benzopyrylium skeleton, determine the types of anthocyanins present. The main anthocyanins detected in grape skin are Cyanidin-3-*O*-glucoside (Cy-3-*O*-glc), Delphinidin-3-*O*-glucoside (Dp-3-*O*-glc), Peonidin-3-*O*-glucoside (Pn-3-*O*-glc), Malvidin-3-*O*-glucoside (Mv-3-*O*-glc), and Petunidin-3-*O*-glucoside (Pt-3-*O*-glc) [13,18,19] (Figure 1). This composition varies with the variety, maturity, climate conditions, viticultural practices, and the production area of grapes [19]. Anthocyanins, like other flavonoids with poly-hydroxy B-ring substitutions, have been found to be free radical scavengers or antioxidants by the DPPH and ABTS radical scavenging methods [20,21]. In recent years, theoretical calculations, particularly the density functional theory (DFT) method, have been utilized to elucidate the structure–activity relationships of phenolic compounds and explore the radical scavenging mechanisms of phenolic compounds [22,23,24,25,26,27,28,29]. It is commonly believed that the reactions between phenolic compounds and reactive oxygen species proceed through three well-established mechanisms: (i) the hydrogen atom transfer (HAT), (ii) the single electron transfer, followed by proton transfer (SET-PT), and (iii) the sequential proton loss electron transfer (SPLET) [30,31,32]. Furthermore, for polyphenolic molecules containing catechol and/or guaiacyl moieties, the phenoxyl radicals generated in the first H^+^/e^−^ reaction may proceed in a second H^+^/e^−^ reaction, which traps the radicals [23,33]. This molecular-level comprehension contributes to the clarification of structure–activity relationships and determines the chemical reactions involved in the antioxidant activity. Furthermore, natural anthocyanins have been found to display inhibitory effects against XO in in vitro and in vivo experiments [34,35]. The anthocyanins can bind to the hydrophobic cavity of XO, cause structural changes to the XO, and affect the functions of enzymes, thereby reducing the catalytic activity of XO and inhibiting the formation of ROS.

Systematic density functional theory calculations were performed in this study to elucidate the antioxidant efficacy of the Cy-3-*O*-glc, Dp-3-*O*-glc, Pn-3-*O*-glc, Mv-3-*O*-glc, and Pt-3-*O*-glc extracted from grape skin. The thermodynamic descriptors related to the three main radical scavenging mechanisms were calculated in order to characterise and compare the antioxidative capacities of the investigated compounds. Moreover, the reaction kinetics of Pt-3-*O*-glc with ^•^OH radical were investigated to gain insights into their mechanisms of action. In addition to these, molecular docking analyses have been conducted to evaluate the inhibitory effects of the five anthocyanins against the XO enzymes. The results obtained are important for the utilisation of grape skin and the development of effective antioxidant therapies.

## 2. Computational Section

The lowest energy conformations of the Cy-3-*O*-glc, Dp-3-*O*-glc, Pn-3-*O*-glc, Mv-3-*O*-glc, and Pt-3-*O*-glc were searched by using the Molclus program in the present work [36]. Subsequently, fully geometrical optimisation and vibrational frequency calculations for these investigated compounds were performed at the M06-2X level, in combination with the 6-311 + G(d,p) basis set, using the Gaussian 16 package [37,38,39]. The solvent effect was considered using the SMD continuum solvation model for all the investigated species [40]. Unrestricted calculations were adopted for open-shell systems. The natural bonding orbital (NBO) analyses were completed by the NBO 6.0 program [41]. The transition states, pre-complex (RC), post-complex (PC), and products of the reaction, between the Pt-3-*O*-glc molecules and ^•^OH radical, were optimised and computed at the same level. Intrinsic reaction coordinate (IRC) calculations were carried out to ensure that the discovered transition states connect reactants and products along the path of the reaction minimum energy [42]. The reaction’s enthalpies, as well as Gibbs free energies, were computed at 298.15 K and 1 M standard state.

### 2.1. Thermodynamic Parameters

The thermodynamic parameters, bond dissociation enthalpy (BDE), ionisation potential (IP), proton dissociation enthalpy (PDE), proton affinity (PA), and electron transfer enthalpy (ETE) related to the three radical scavenging mechanisms were calculated. The calculated equations for these are listed below, as follows:

HAT: BDE = H(ArO^•^) + H(H^•^) − H(ArOH) (1)

SET-PT: IP = H(ArOH^•+^) + H(e^−^) − H(ArOH) (2)

PDE = H(ArO^•^) + H(H^+^) − H(ArOH^•+^) (3)

SPLET: PA = H(ArO^−^) + H(H^+^) − H(ArOH) (4)

ETE = H(ArO^•^) + H(e^−^) − H(ArO^−^) (5)

In Equations (1)–(5), H(ArO^•^), H(ArO^−^), and H(ArOH^•+^) are the enthalpies of the radical, anion, and radical cation of the phenolic compounds, respectively. H(H^•^), H(H^+^), and H(e^−^) were collected from previous studies [43,44]. The solvation enthalpies of H(H^+^) and H(e^−^) were calculated based on reports in the literature [45].

### 2.2. Kinetic Parameters

The rate constants for HAT pathways were computed by conventional transition state theory (TST) [46], utilising the KiSThelP 2019 program according to Equation (6) [47].

(6) $$k_{TST}=\sigma_{k}\frac{k_{B}T}{h}exp(-\frac{\Delta G^{\#}}{RT})\;$$

where *σ*, *k_B_*, *T*, *h*, Δ*G^#^* are the reaction path degeneracy, Boltzmann constant, temperature, Planck constant, and Gibbs free energy of activation, respectively. The *k* represents tunnelling corrections, calculated according to the Wigner method [48].

### 2.3. Molecular Docking Studies 

Molecular docking analyses have been carried out to investigate the inhibitory effects of anthocyanin molecules against the XO enzyme. The structure of XO with PDB ID (3NVY) was downloaded from the RCSB PDB (https://www.rcsb.org (accessed on 1 April 2024)) [49]. Then, the water molecules were removed, while hydrogen atoms and charges were added to the protein. The structures of ligands (five anthocyanins) were obtained from the most stable conformers mentioned above. Autodock Tools (version 1.5.6) were used to generate PDBQT files, which were then used for docking in AutoDock software (version 4.2.6) [50]. The grid box was centred on the coordinates of X: 39.06, Y: 21.90, and Z: 20.22 with dimensions of 40 Å × 40 Å × 40 Å along the *x*-, *y*-, and *z*-axis, with the scaling factor of 0.375. Molecular docking was carried out by using the defined Lamarckian Genetic Algorithm method to represent the potential energy surface of the ligand–protein interaction. The obtained conformations are visualised with the PyMol package (Version 2.6.0a0) [51]. Two-dimensional diagrams of the anthocyanins interacting with the XO are depicted with Discovery Studio 2024 software.

## 3. Results and Discussion

### 3.1. Conformational Analysis

The antiradical activity of phenolic compounds could be greatly influenced by the structures of the molecule. In order to determine the lowest energy conformation for further studies, we first performed the conformational search for the Cy-3-*O*-glc, Dp-3-*O*-glc, Pn-3-*O*-glc, Mv-3-*O*-glc, and Pt-3-*O*-glc using the Molclus program and the XTB code. Subsequently, the low-lying isomers were re-optimised at the M06-2X level, with the vibrational frequencies confirmed by the absence of imaginary frequencies. Figure S1 shows the optimised lowest-lying conformers of the five anthocyanins in the gas phase. As revealed by the dihedral angles (C3-C2-C1′-C2′) between the B-ring and the C-ring, all studied molecules show non-planar geometries. A comparison between the five anthocyanins reveals differences in either the number or position of hydroxyl and/or methoxy groups. As shown in Figure 1, the Dp-3-*O*-glc has five hydroxyl groups distributed among the C3′, C4′, C5′, C5, and C7 sites of the benzopyrylium skeleton. The Pt-3-*O*-glc can be seen as the replacement of the 5′-OH group, with one methoxy in the Dp-3-*O*-glc. Furthermore, studies have been carried out using water and ethanol as solvents. For the studied anthocyanins, there is no significant difference in the structure of the most stable conformation (ArOH) and its corresponding radicals (ArO^•^), anions (ArO^−^), and radical cations (ArOH^•+^) at the same level. Additionally, the molecular orbital analysis indicates that the electron density for HOMO and LUMO orbitals of five anthocyanins are both distributed on the A-, B- and C-ring. It has shown that there is no density distribution of glycoside substituent on the C-ring, hence indicating that OH groups on the C3-glycosyl substituent of the anthocyanins are not easily involved in the reaction (Figure S2). Moreover, detailed NBO analyses on phenolic hydroxyls of the benzopyrylium skeleton for five anthocyanins show that the charge on the hydrogen atom of 4′-OH in Table S1 was almost the highest, indicating that 4′-OH is more prone to react with oxygen radicals compared with 3′-OH, 5′-OH, 5-OH, and 7-OH in the anthocyanins.

### 3.2. Analysis of Radical Scavenging Reactions

The thermodynamic calculations related to the radical-scavenging mechanisms were performed to predict the radical scavenging activity of Cy-3-*O*-glc, Dp-3-*O*-glc, Pn-3-*O*-glc, Mv-3-*O*-glc, and Pt-3-*O*-glc. 

#### 3.2.1. HAT Mechanism

The bond dissociation enthalpy (BDE) is the critical descriptor for assessing the radical scavenging activity of antioxidants through the HAT mechanism. The lower BDE is more inclined to donate a H-atom, which indicates the higher radical scavenging capacity of the investigated compound. At the M06-2X/6-311 + G(d,p) level, the calculated BDEs for Cy-3-*O*-glc, Dp-3-*O*-glc, Pn-3-*O*-glc, Mv-3-*O*-glc, and Pt-3-*O*-glc in the gas phase and the solvents (water and ethanol) are summarised in Table 1. It can be observed that the solvent effect has little influence on the BDEs of OH on the A ring, while its impact on the BDE of OH on the B ring is relatively large. The BDE values of 4′-OH for the studied anthocyanins were almost the lowest among all these sites of O-H bonds, except Cy-3-*O*-glc, showing that the cleavage of 4′-OH was more prone to transfer hydrogen atom to radical among all hydroxyl groups, which is consistent with previous research [52,53,54]. For example, Ma et al. explored four anthocyanins from purple potatoes [54] and indicated that 4′-OH has the lowest BDE values compared with other phenolic hydroxyls in the anthocyanins. Dudek et al. found that homolytic fission from the C4′ hydroxyl group is most feasible among nearly all anthocyanins [52]. In addition, the sequence of the 4′-OH BDE values of five anthocyanin molecules in the gas phase is arranged as follows: Pt-3-*O*-glc (86.2 kcal/mol) < Dp-3-*O*-glc (86.7 kcal/mol) < Mv-3-*O*-glc (87.4 kcal/mol) < Cy-3-*O*-glc (89.8 kcal/mol) < Pn-3-*O*-glc (93.4 kcal/mol). Evidently, the BDEs of 4′-OH in Mv-3-*O*-glc and Pt-3-*O*-glc are significantly lower than that of Pn-3-*O*-glc (average of about 6.6 kcal/mol) due to the replacement of -H with -OH/-OCH_3_. Moreover, compared with Pn-3-*O*-glc and Mv-3-*O*-glc molecules, the substitution of the methoxy group in C3′ positions of Mv-3-*O*-glc decreases the BDEs values of 4′-OH places, but does not significantly affect 5-OH and 7-OH. 

#### 3.2.2. SET-PT Mechanism

In the SET-PT mechanism, electron transfer from the antioxidant species is followed by proton transfer. This mechanism is governed by ionisation potential (IP) and proton dissociation enthalpy (PDE). Table 1 summarises the calculated IP and PDE values involved in the SET-PT pathway in different phases for five anthocyanins. The IP is a critical parameter that shows the range of electron donation. Compared to the BDEs, it was found that solvents produce an obvious effect on the IPs. Compared with the gas phase, the IPs in the water and ethanol solvents decreased significantly by about 101.5 and 105.6 kcal/mol, respectively. These results indicate that polar solvents are beneficial for abstracting electrons from the investigated anthocyanins. The PDE is an essential thermodynamic parameter for characterising the second step of the SET-PT mechanism. As the cation radicals are charged and sensitive to environmental polarity, the PDEs notably decrease with changes to the environment. Similar to the BDE values, the C4′ hydroxyl group is the most active site of all anthocyanin molecules because it has the lowest PDE values in both the solvents and the gas phase. 

#### 3.2.3. SPLET Mechanism

The SPLET mechanism proceeds via two-step reactions. The PA and ETE parameters were calculated to explore the feasibility of the SPLET pathway for five anthocyanins. The calculation results are listed in Table 1. The sequence of the PAs of the studied anthocyanins in the medium is gas >> solvents (water and ethanol). For example, the PAs of 4′-OH in Pt-3-*O*-glc decrease from 251.7 kcal/mol in the gas phase to 23.8 and 22.5 kcal/mol in the water and ethanol phase, respectively, indicating that the solvents appear to favour the deprotonation. It is clearly observed that the PAs of the C5 and C7 hydroxyl group on the A ring are lower than that of the C3′, C4′, and C5′ hydroxyl group on the B ring for all of the studied molecules. As far as the ETEs are concerned, it can be observed that Pt-3-*O*-glc has the lowest ETE values compared with that of the other four anthocyanins in the gas and solvents phases, which are consistent with the sequence of BDE values.

As mentioned above, the preferred mechanism for molecules is mainly determined by the values of BDE, IP, and PA, in which the IP and PA define the first step and the predominant step of the SET-PT and SPLET mechanisms, respectively. By comparison, it can be found that the BDEs are always lower than the IPs and PAs in the gas phase corresponding to the five molecules, which shows that the HAT mechanism is thermodynamically preferable for trapping radicals in the gas phase. However, these anthocyanins have the lowest PAs in the solvents phase compared with their BDEs and IPs, illustrating that the antioxidant process inclines to the SPLET mechanism in the solvents phase. The above findings are consistent with the predominant mechanism of delphinidin and derivatives for antioxidant activity in different phases [52].

#### 3.2.4. Double HAT Mechanism 

**Figure 2:**
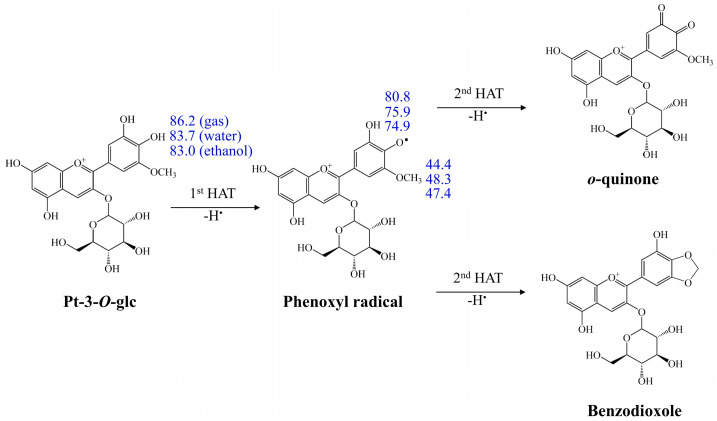
Double HAT mechanism of Pt-3-*O*-glc in the gas phase and solvents (unit: kcal/mol).

Figure 2 presents the double HAT mechanism for the representative Pt-3-*O*-glc in the gas and solvents phase. As mentioned above, the C4′ hydroxyl group is the preferred site for donation of H atoms. After the first HAT process at 4′-OH in Pt-3-*O*-glc, the phenoxyl 4′-O radical can undergo the second H^+^/e^−^ reaction using the C5′-OCH_3_ group to form a stable benzodioxole, or by using the C3′-OH group to generate o-quinone. It can be observed that the BDEs for the formation of benzodioxole are only 44.4, 48.3, and 47.4 kcal/mol in the gas, water, and ethanol phases, respectively, which is similar to previous reports and makes it feasible for trapping radicals [23]. Additionally, the single states of produced o-quinone are 80.8, 75.9, and 74.9 kcal/mol in the gas, water, and ethanol phases, respectively, which are lower than the corresponding BDEs of the 3′-OH in the first HAT reaction. Our calculations indicate that the energy cost of forming benzodioxole is considerably reduced compared to the energy cost of forming o-quinone in the second HAT process. Accordingly, the thermodynamically favoured product is benzodioxole via the double HAT mechanism.

### 3.3. Kinetic Study

**Figure 3:**
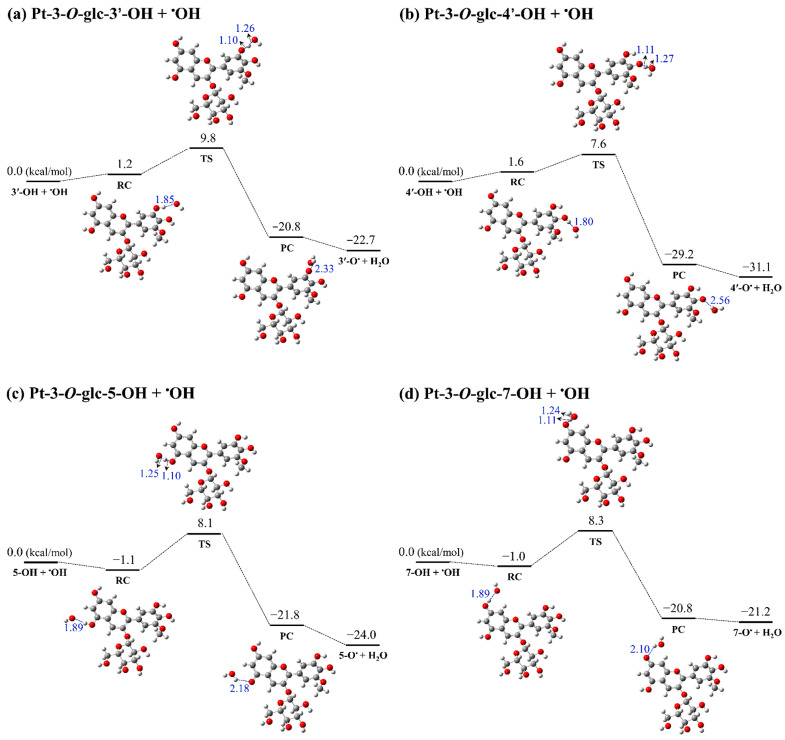
The PESs of reaction between Pt-3-*O*-glc and ^•^OH via HAT pathway in the gas phase. The distances are shown in blue (unit: Å). The gray, red, and white balls represent the elements C, O, and H, respectively.

The rate constant is another major criterion for identifying the free radical scavenging activity of the antioxidants. Based on the calculated thermodynamic parameters, Pt-3-*O*-glc possesses the strongest antioxidant capacity among the five anthocyanin molecules. In this study, the reaction pathway between Pt-3-*O*-glc and hydroxyl radicals (^•^OH) has been systematically calculated via the HAT mechanism. The potential energy surfaces (PESs) of the reaction between ^•^OH and Pt-3-*O*-glc were calculated. Table 2 and Figure 3 summarize Pt-3-*O*-glc’s reaction kinetics and thermodynamics with the ^•^OH radical. All calculations were performed in the gas phase at the M06-2X/6-311+G(d,p) level. As can be seen in Figure 3, the reaction energy barriers of the O3′, O4′, O5, and O7 positions of Pt-3-*O*-glc with the ^•^OH following the HAT mechanism are 9.8, 7.6, 8.1, and 8.3 kcal/mol, respectively, which indicates shallow barriers when H atom transfer reacts with the ^•^OH for the HAT reaction. Compared to the reactant complexes, it was found that the reactions between the O3′, O4′, O5, and O7 positions of Pt-3-*O*-glc and the ^•^OH radical are all exergonic, with Gibbs free energy decreasing by 22.7, 31.1, 24.0, and 21.2 kcal/mol in the gas phase, respectively. It can be observed that the lowest ΔrG value and barrier of Pt-3-*O*-glc scavenging ^•^OH radical are located at 4′-OH in the gas phase, which is probably a more favorable site for radical attacks. For the optimised structures of TSs, it can be observed that the distances for breaking O···H bonds and forming H···O bonds are in the range of 1.10–1.11 and 1.24–1.27 Å, respectively. Furthermore, it is clearly shown that the reaction between Pt-3-*O*-glc-4′-OH and ^•^OH has the highest rate constant (5.72 × 10^9^ M^−1^ s^−1^). It suggests that Pt-3-*O*-glc-4′-OH has the highest antioxidant activity in the HAT mechanism.

### 3.4. Molecular Docking

**Figure 4:**
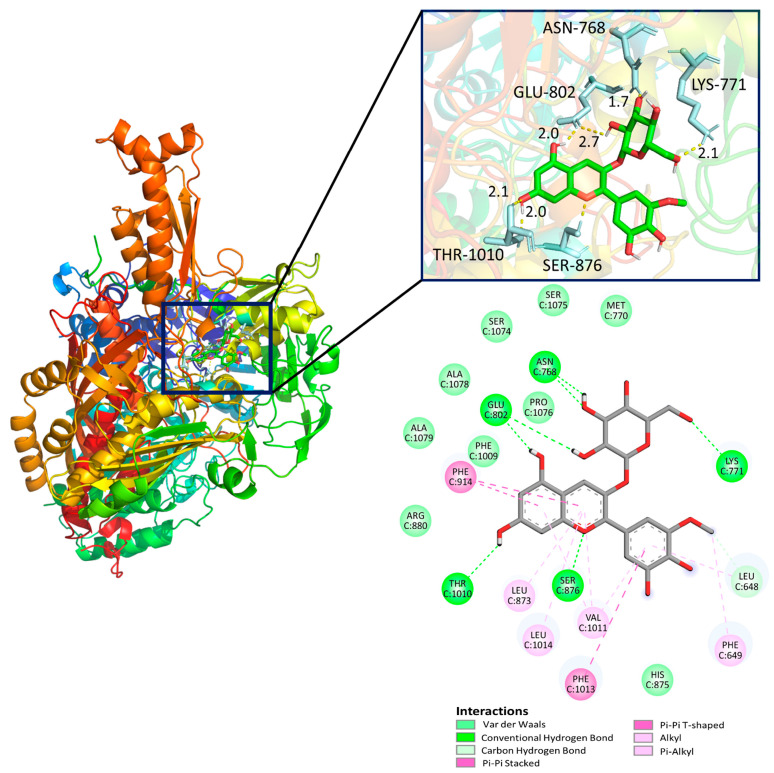
The 3D and 2D interactions of the Pt-3-*O*-glc with XO, along with the corresponding binding poses.

Molecular docking is a computational approach that is used to identify the interaction between the ligand (small molecule) and the active sites of the protein in the lowest possible energy state. XO is a superoxide-producing enzyme for the catalysis of xanthine to hypoxanthine and hypoxanthine to uric acid, with more than 1300 amino acid residues. In this study, we performed molecular docking studies of five compounds with the XO enzyme. The 3D and 2D interactions of the Pt-3-*O*-glc with XO, along with their corresponding binding poses, are illustrated in Figure 4. The 2D interactions of the Cy-3-*O*-glc, Dp-3-*O*-glc, Pn-3-*O*-glc, and Mv-3-*O*-glc with XO are given in Figure S3, alongside the binding-free energies listed in Table S2. It can be noted that Pt-3-*O*-glc had hydrogen-bonding interactions with the residues ASN768, LYS771, GLU802, SER876, and THR1010 and showed a binding energy of -9.11 kcal/mol. The results indicated that the glycosidic group of Pt-3-*O*-glc makes significant contributions to the formation of hydrogen-bonding interactions and the stabilisation of inhibitor conformations. It can be observed that residues Phe914 and Phe1013 are related to π-π stacking interaction with the aromatic ring of Pt-3-*O*-glc, which contributes to enhancing the stability of the complex. Additionally, the other four anthocyanin molecules showed similar binding poses and binding energy to Pt-3-*O*-glc, which suggests that these anthocyanins compounds could stably bind to the active site of XO. Significantly, Xie et al. investigated the binding modes and interactions between delphinidin-3-*O*-sambubioside and XO and found that the delphinidin-3-*O*-sambubioside formed hydrogen bonds with several residues, including LEU648, ASN768, LYS771, GLU802, and SER876, exhibiting a good bonding ability for XO [35]. Meanwhile, these anthocyanins prevent other compounds from entering the active pocket of XO and inhibit the catalytic pathway from the activation of the XO enzyme generating ROS. Therefore, it could be concluded that anthocyanin compounds have significant inhibitory potential against xanthine oxidase.

## 4. Conclusions

In this paper, DFT calculations were performed to assess the radical scavenging activity of five anthocyanins extracted from grape skin. The three well-established mechanisms, including HAT, SET-PT, SPLET as well as a double HAT mechanism, were studied in detail in the gas and solvent phases. Among the studied anthocyanins, Pt-3-*O*-glc had the strongest antioxidant capacity, with the most active site of 4′-OH. It was found that the HAT mechanism is thermodynamically preferable for trapping radicals in the gas phase, while the SPLET is remarkably favourable in the solvents phase. The present work indicates that the chemical nature and the reaction environments of the antioxidant molecule play an important role in the antioxidant defence mechanism. The results also reveal the possibility of using the double HAT mechanism for the Pt-3-*O*-glc compound, whose thermodynamically favoured product is benzodioxole. The reaction kinetics of Pt-3-*O*-glc with ^•^OH radical results show that the reaction of ^•^OH into the 4′-OH of Pt-3-*O*-glc is more favourable than other reactions with a lower energy barrier (7.6 kcal/mol) and higher rate constants (5.72 × 10^9^ M^−1^ s^−1^). The molecular docking results revealed that these anthocyanin compounds can inhibit ROS formation by interacting with the xanthine oxidase. The above results highlight the antioxidant mechanism of the anthocyanins and their inhibitory mechanisms against XO. We believe that our findings will provide a theoretical foundation for the utilisation of grape skin and the development of new food antioxidants.

## Figures and Tables

**Table 1:** The calculated BDE, IP, PDE, PA, and ETE values for the five anthocyanins in the gas phase and solvents (unit: kcal/mol).

Mechanism	HAT			SET-PT						SPLET					
	BDE			IP			PDE			PA			ETE		
Compounds	Gas	Water	Ethanol	Gas	Water	Ethanol	Gas	Water	Ethanol	Gas	Water	Ethanol	Gas	Water	Ethanol
**Cy-3-*O*-glc**				244.5	139.7	135.9									
4′-OH	94.0	88.9	88.2				162.8	3.1	−0.7	255.7	25.8	25.0	151.6	117.0	110.3
5′-OH	89.8	87.5	86.8				158.6	1.8	−2.1	268.0	30.1	30.2	135.1	111.4	103.6
5-OH	94.2	93.9	93.1				163.0	8.1	4.2	247.0	23.0	21.7	160.5	124.8	118.4
7-OH	96.7	96.6	95.7				165.5	10.8	6.8	246.3	22.4	20.9	163.7	128.1	121.8
**Dp-3-*O*-glc**				243.2	139.4	135.6									
3′-OH	94.7	89.6	89.2				164.8	4.2	0.7	276.7	31.6	32.6	131.3	112.0	103.6
4′-OH	86.7	84.0	83.4				156.9	−1.4	−5.2	250.2	23.1	22.0	149.8	114.9	108.4
5′-OH	90.6	88.5	87.6				160.7	3.1	−0.9	268.0	29.8	29.9	135.9	112.7	104.8
5-OH	94.1	93.9	93.2				164.2	8.4	4.7	246.9	22.9	21.6	160.5	124.9	118.7
7-OH	96.9	96.6	96.1				167.0	11.2	7.5	246.3	22.5	20.9	163.9	128.1	122.2
**Pn-3-*O*-glc**				240.0	138.5	134.5									
4′-OH	93.4	88.6	88.0				166.8	4.1	0.5	257.2	26.5	25.6	149.6	116.0	109.4
5-OH	93.9	93.7	93.0				167.3	9.2	5.5	247.7	23.0	21.6	159.6	124.6	118.5
7-OH	96.4	96.9	95.8				169.7	12.4	8.3	247.2	22.6	21.0	162.5	128.2	121.8
**Mv-3-*O*-glc**				234.8	138.9	134.0									
4′-OH	87.4	86.3	85.0				165.9	1.4	−2.0	255.5	24.9	23.7	145.3	115.4	108.3
5-OH	93.9	93.3	93.0				172.4	8.3	6.0	248.2	22.8	21.2	159.0	124.4	118.9
7-OH	96.4	97.0	95.5				174.9	12.1	8.5	247.8	22.3	20.6	162.0	128.6	122.0
**Pt-3-*O*-glc**				239.6	138.1	134.3									
3′-OH	94.6	89.6	89.1				168.4	5.4	1.8	278.0	32.7	33.2	130.0	110.9	102.9
4′-OH	86.2	83.7	83.0				160.0	−0.4	−4.3	251.7	23.8	22.5	147.8	113.9	107.5
5-OH	94.0	93.8	93.2				167.7	9.7	5.9	247.7	22.8	21.5	159.6	125.0	118.8
7-OH	96.7	96.8	96.0				170.4	12.7	8.7	247.3	22.2	20.8	162.7	128.6	122.3 qstat

**Table 2:** The calculated activation (ΔG^≠^), Gibbs free energies (ΔG), and rate constants (*k*) for the reaction between Pt-3-*O*-glc and ^•^OH radical in the gas phase at 298.15 K.

Reactions	ΔG (kcal/mol)	ΔG^≠^ (kcal/mol)	*k* (M^−1^ s^−1^)
Pt-3-*O*-glc-3′-OH + ^•^OH	−22.7	9.8	7.45 × 10^7^
Pt-3-*O*-glc-4′-OH + ^•^OH	−31.1	7.6	5.72 × 10^9^
Pt-3-*O*-glc-5-OH + ^•^OH	−24.0	8.1	1.33 × 10^9^
Pt-3-*O*-glc-7-OH + ^•^OH	−21.2	8.3	9.31 × 10^8^

## Data Availability

Data are contained within this article.

## Supplementary Materials

The following supporting information can be downloaded at: https://www.mdpi.com/article/10.3390/antiox13091117/s1, Figure S1: optimized structures of the Cy-3-*O*-glc, Dp-3-*O*-glc, Pn-3-*O*-glc, Mv-3-*O*-glc, and Pt-3-*O*-glc at M06-2X/6-311+G(d,p) level in the gas phase; Figure S2: the electron density distribution and energies of the HOMO and LUMO for Cy-3-*O*-glc, Dp-3-*O*-glc, Pn-3-*O*-glc, Mv-3-*O*-glc, and Pt-3-*O*-glc calculated at the M06-2X level in the gas phase; Figure S3: the 2D interactions of the Cy-3-*O*-glc, Dp-3-*O*-glc, Pn-3-*O*-glc, and Mv-3-*O*-glc with XO along with the corresponding binding poses; Table S1: calculated natural bond orbital (NBO) charges on the hydrogen atoms of phenolic hydroxyls for Cy-3-*O*-glc, Dp-3-*O*-glc, Pn-3-*O*-glc, Mv-3-*O*-glc, and Pt-3-*O*-glc; Table S2: binding energy (kcal/mol) of Cy-3-*O*-glc, Dp-3-*O*-glc, Pn-3-*O*-glc, and Mv-3-*O*-glc with the XO enzyme; Table S3: optimized coordinates (x, y, z) of Cy-3-*O*-glc, Dp-3-*O*-glc, Pn-3-*O*-glc, Mv-3-*O*-glc, and Pt-3-*O*-glc at M06-2X level in the gas phase.
