# Supplementary material for: Computational Insights into the Radical Scavenging Activity and Xanthine Oxidase Inhibition of the Five Anthocyanins Derived from Grape Skin
Source: Antioxidants (Basel). 2024 Sep 15;13(9):1117. doi: 10.3390/antiox13091117 (PMC11428504; doi:10.3390/antiox13091117)
Supplement: Supplementary file 1 [file antioxidants-13-01117-s001.zip › antioxidants-3155091-supplementary.pdf]

Electronic Supporting Information

Computational Insights into the Radical Scavenging Activity and Xanthine Oxidase Inhibition of the Five Anthocyanins Derived from Grape Skin

Xiao-Qin Lu*, Jindong Li, Bin Wang and Shu Qin

Shanxi Center for Testing of Functional Agro-Products, Shanxi Agricultural University, Taiyuan 030031, China

**E-mails:* luxiaoqin@sxau.edu.cn

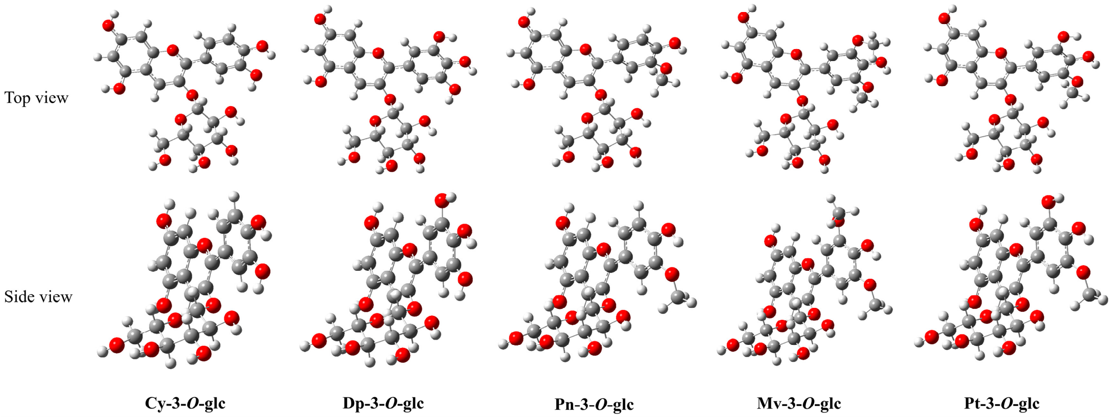

Figure S1. Optimized structures of the Cy-3-*O*-glc, Dp-3-*O*-glc, Pn-3-*O*-glc, Mv-3-*O*-glc, and Pt-3-*O*-glc at M06-2X/6-311+G(d,p) level in the gas phase.

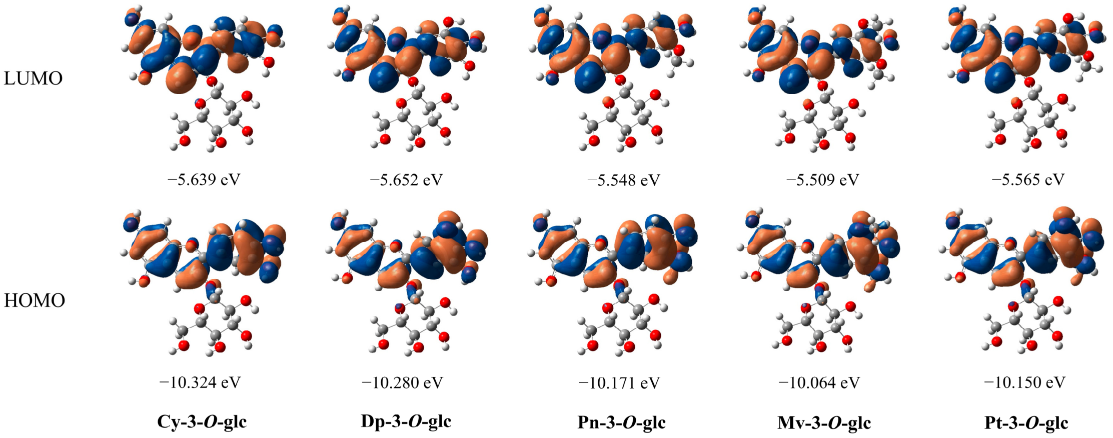

Figure S2. The electron density distribution and energies of the HOMO and LUMO for Cy-3-*O*-glc, Dp-3-*O*-glc, Pn-3-*O*-glc, Mv-3-*O*-glc, and Pt-3-*O*-glc calculated at the M06-2X level in the gas phase.

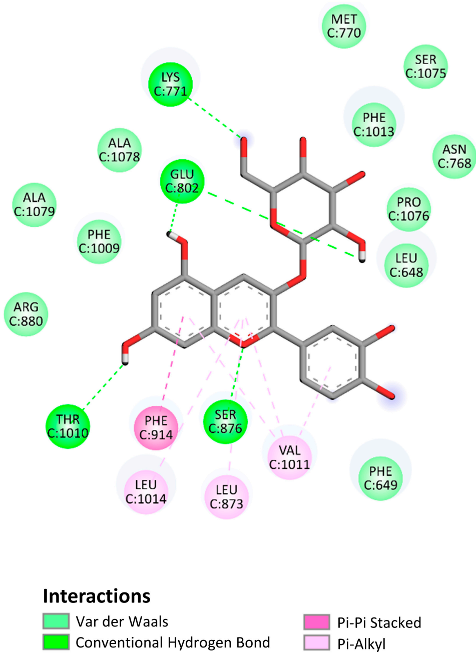

Cy-3-*O*-glu

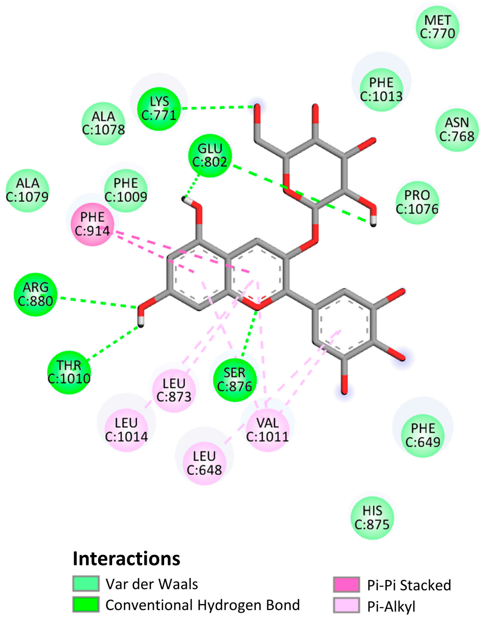

Dp-3-*O*-glu

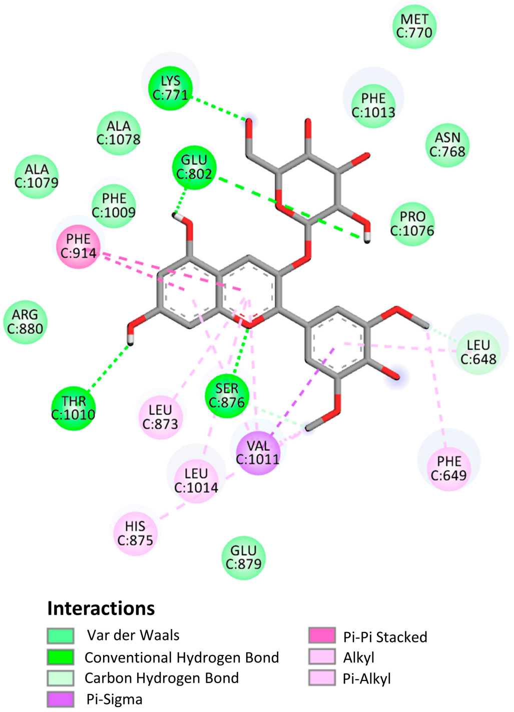

Mv-3-*O*-glu

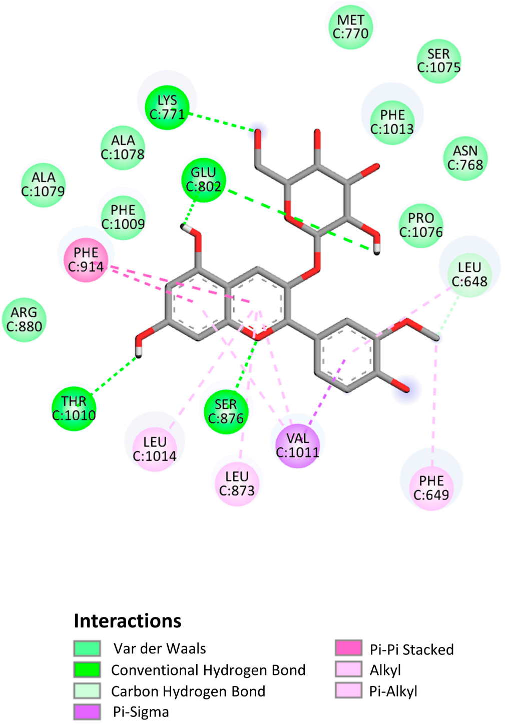

Pn-3-*O*-glu

Figure S3. The 2D interactions of the Cy-3-*O*-glc, Dp-3-*O*-glc, Pn-3-*O*-glc, and Mv-3-*O*-glc with XO along with the corresponding binding poses.

Table S1. Calculated natural bond orbital (NBO) charges on the hydrogen atoms of phenolic hydroxyls for Cy-3-*O*-glc, Dp-3-*O*-glc, Pn-3-*O*-glc, Mv-3-*O*-glc, and Pt-3-*O*-glc.

Reactions	Cy-3-*O*-glc	Dp-3-*O*-glc	Pn-3-*O*-glc	Mv-3-*O*-glc	Pt-3-*O*-glc
**3′-OH**		0.503			0.502
**4′-OH**	0.509	0.518	0.509	0.512	0.518
**5′-OH**	0.514	0.513			
**5-OH**	0.500	0.500	0.499	0.499	0.499
**7-OH**	0.494	0.495	0.494	0.494	0.495

Table S2. Binding energy (kcal/mol) of Cy-3-*O*-glc, Dp-3-*O*-glc, Pn-3-*O*-glc, and Mv-3-*O*-glc with the XO enzyme.

	Binding Free Energy (kcal/mol)
**Cy-3-*O*-glc**	-8.62
**Dp-3-*O*-glc**	-8.57
**Pn-3-*O*-glc**	-9.38
**Mv-3-*O*-glc**	-9.62

Table S3. Optimized coordinates (x, y, z) of Cy-3-*O*-glc, Dp-3-*O*-glc, Pn-3-*O*-glc, Mv-3-*O*-glc, and Pt-3-*O*-glc at M06-2X level in the gas phase.

Cy-3-*O*-glc

C	1.86197300	-2.59660400	0.82811700
C	3.28736700	-2.68352600	0.26646100
C	3.74985500	-1.29023400	-0.12164200
C	2.77331900	-0.60960700	-1.06410200
C	1.40399800	-0.60773900	-0.40554200
O	0.46483600	-0.11508400	-1.33994000
C	-5.62567500	-0.30281300	0.36666400
C	-3.39636800	0.41873400	0.03479800
C	-4.66790200	0.69705900	0.50505100
C	-3.04232700	-0.80303300	-0.56128500
C	-5.32257900	-1.54872100	-0.22097200
C	-1.72085700	-0.96654400	-1.00366300
C	-0.80493100	0.05157400	-0.86676900
C	-1.19584100	1.26748500	-0.26304200
H	-6.11408000	-2.28456400	-0.29814500
H	-1.39986800	-1.89887500	-1.45369700
O	1.01451800	-1.91943200	-0.10011700
O	3.21489200	0.71246000	-1.32926600
H	4.14877000	0.62075700	-1.56546300
O	4.99137000	-1.29980700	-0.78527800
H	5.63079500	-1.75702500	-0.22689700
O	4.22498900	-3.12908100	1.21466500
H	3.92245300	-3.97248900	1.57440300
C	1.21896900	-3.96250400	1.01333600
H	0.24078600	-3.84338800	1.48907000
C	-4.05361600	-1.79947400	-0.67819000
O	-3.66555800	-2.95002800	-1.24876600
H	-4.39966600	-3.56904000	-1.32624200
O	-2.45853600	1.38600800	0.15468400
O	-6.88833300	-0.15698700	0.77580000
H	-7.04329600	0.71191400	1.16171000
C	-0.36811300	2.40670400	0.01724900
C	0.88139400	2.55991800	-0.61876400
C	-0.77287300	3.35786200	0.97638200
C	1.72265500	3.57610600	-0.24644800
H	1.20123100	1.89573400	-1.40506200
C	0.06079000	4.40264200	1.31654100
H	-1.72964000	3.25591200	1.47023600
C	1.32001900	4.51692900	0.72167000

O	2.13273000	5.50774200	1.07654400
H	2.96673900	5.43401800	0.59071400
H	-4.89124200	1.65807200	0.94989600
H	1.39826800	0.01926100	0.50009200
H	2.69415500	-1.17955500	-1.99777500
H	3.82027500	-0.69388400	0.80123000
H	3.28168500	-3.32669300	-0.62611600
H	1.89131300	-2.05755600	1.78548500
O	2.09524000	-4.73518400	1.81536800
H	1.72367300	-5.60725200	1.96743700
H	1.08244000	-4.41447600	0.02440500
O	2.96066300	3.75452600	-0.76781800
H	3.24931900	2.92767900	-1.17809800
H	-0.22415900	5.13511000	2.06068800

Dp-3-*O*-glc

C	1.97665800	-2.66397500	0.91289500
C	3.40631600	-2.72878800	0.35940100
C	3.81900500	-1.34235200	-0.10241400
C	2.82246000	-0.74987400	-1.08279700
C	1.45096100	-0.76395700	-0.42880100
O	0.49808800	-0.35896900	-1.39106000
C	-5.58885200	-0.67383800	0.30478800
C	-3.38667700	0.10938300	-0.06153300
C	-4.67005200	0.36751300	0.38732900
C	-2.98347300	-1.13200500	-0.58298700
C	-5.23658600	-1.94058600	-0.20788100
C	-1.65551300	-1.27091300	-1.00956400
C	-0.77790500	-0.21141300	-0.93001600
C	-1.21870500	1.02003700	-0.40152300
H	-6.00030200	-2.70827100	-0.24412300
H	-1.29648900	-2.21503100	-1.40284100
O	1.10897500	-2.07102800	-0.05349300
O	3.21528800	0.57175400	-1.41693100
H	4.15291200	0.50329500	-1.64557500
O	5.06333000	-1.34214400	-0.76095500
H	5.71548000	-1.74686700	-0.17735800
O	4.35640100	-3.08803200	1.33153300
H	4.08266900	-3.92016600	1.73748300
C	1.38367300	-4.04029000	1.17243000
H	0.39872400	-3.93103100	1.63644700
C	-3.95719200	-2.17099600	-0.64484600
O	-3.52326200	-3.33754000	-1.14518900

H	-4.23383400	-3.98648100	-1.19094100
O	-2.48608500	1.11482100	0.00431100
O	-6.85781500	-0.55207300	0.70045500
H	-7.04636700	0.33053200	1.03752300
C	-0.43769300	2.20998400	-0.18309300
C	0.80388900	2.36699400	-0.82302200
C	-0.90458300	3.19250500	0.71073500
C	1.59355900	3.45111600	-0.51175900
H	1.15970400	1.66763500	-1.56061100
C	-0.12158700	4.29726000	0.98378700
H	-1.85500400	3.09670900	1.21690700
C	1.14028800	4.42323400	0.38244100
O	1.87695600	5.49406200	0.71117500
H	2.72933900	5.46227900	0.25473400
H	-4.93053200	1.34403300	0.77461000
H	1.41735200	-0.08923300	0.44099700
H	2.76912300	-1.37169400	-1.98469400
H	3.86316700	-0.69488600	0.78703200
H	3.42717100	-3.41873500	-0.49731700
H	1.98186500	-2.07197800	1.83889100
O	2.28278800	-4.73316100	2.02083000
H	1.94355000	-5.60922500	2.21864600
H	1.27032900	-4.55229100	0.21016500
O	2.82823200	3.65465000	-1.03177800
H	3.15539700	2.81956000	-1.39338400
O	-0.55740200	5.23124300	1.84886100
H	0.10384400	5.92895600	1.93992200

Pn-3-*O*-glc

C	1.04625700	-3.20884200	0.71510200
C	2.49635100	-3.52387500	0.32040000
C	3.27263300	-2.22385400	0.19872800
C	2.59122600	-1.25304200	-0.74845100
C	1.17857300	-1.03552300	-0.24644000
O	0.49811700	-0.21616200	-1.17664100
C	-5.60281900	0.56424200	0.31053500
C	-3.27219800	0.90476600	0.07902500
C	-4.48969200	1.36225200	0.55359000
C	-3.11926700	-0.30449100	-0.61914000
C	-5.50468400	-0.65990000	-0.38356900
C	-1.83075400	-0.66898100	-1.03838200
C	-0.75438900	0.15170200	-0.78359500
C	-0.94773300	1.37151800	-0.10431000

H	-6.41010000	-1.23433100	-0.53904400
H	-1.66152500	-1.60810300	-1.55167200
O	0.48573600	-2.25389100	-0.18630000
O	3.26933400	-0.01867100	-0.77018000
H	4.21310800	-0.22503900	-0.78667200
O	4.57557200	-2.41839300	-0.29669100
H	5.02093900	-3.05872600	0.27003900
O	3.17731400	-4.26935100	1.29952100
H	2.66750000	-5.06688700	1.48940000
C	0.13325600	-4.42069800	0.60968500
H	-0.86483900	-4.15747400	0.97348100
C	-4.28580900	-1.09102500	-0.84233400
O	-4.08856800	-2.23963300	-1.50773600
H	-4.91757600	-2.70951200	-1.64905900
O	-2.18583700	1.68002000	0.29410500
O	-6.83295200	0.89603700	0.71134100
H	-6.84422400	1.74121900	1.17332200
C	0.04254800	2.35170600	0.25060200
C	1.25823700	2.46661100	-0.46689500
C	-0.23040200	3.23436600	1.31294500
C	2.15054800	3.45551500	-0.12293900
H	1.48273500	1.79724000	-1.28328100
C	0.68869400	4.19455500	1.67952100
H	-1.15770800	3.14664200	1.86250800
C	1.87882900	4.31731500	0.96586500
O	2.75827100	5.25420000	1.30541800
H	3.51330500	5.21476600	0.70030100
H	-4.55490600	2.30660000	1.07828800
H	1.18537100	-0.54285500	0.73908800
H	2.53430600	-1.69051400	-1.75351700
H	3.31521800	-1.76660600	1.19958700
H	2.49944100	-4.04025800	-0.65104400
H	1.03905900	-2.81620500	1.74165500
O	0.71195000	-5.45499500	1.38675800
H	0.16662800	-6.24408300	1.34686500
H	0.06424900	-4.70756200	-0.44573500
O	3.32591500	3.72123800	-0.73474200
H	0.50950800	4.86936700	2.50635500
C	3.58012600	3.09635700	-1.99133900
H	3.62140200	2.01263600	-1.87882400
H	2.80164300	3.37258100	-2.70767600
H	4.54046100	3.47984900	-2.32479400

Mv-3-*O*-glc

C	2.32460100	2.73512400	0.95209800
C	1.85522800	4.14443500	0.56346300
C	0.36975600	4.10515800	0.24861200
C	0.04757800	3.05518500	-0.79923500
C	0.56408200	1.72456300	-0.29133700
O	0.36563200	0.75615900	-1.30203300
C	2.88844300	-4.85729400	0.16811700
C	1.35795800	-3.07852500	-0.13570000
C	1.59046100	-4.37394400	0.29401400
C	2.35599000	-2.25209500	-0.67813100
C	3.92871700	-4.06905200	-0.36794300
C	1.99939500	-0.95246400	-1.06732900
C	0.70047600	-0.51291200	-0.93314700
C	-0.28064900	-1.37779500	-0.40919700
H	4.91784300	-4.50573200	-0.43765200
H	2.74241600	-0.27072100	-1.46373100
O	1.94510800	1.79472100	-0.05268100
O	-1.34311000	2.96571600	-1.00299100
H	-1.67443400	3.87295400	-1.02800700
O	-0.10698100	5.33084700	-0.25305900
H	0.11766400	6.02299200	0.37937800
O	1.98482000	5.07078600	1.61398200
H	2.89968500	5.06089400	1.92196900
C	3.83922100	2.62296500	1.03309200
H	4.11214500	1.62324700	1.38504100
C	3.67178700	-2.78779600	-0.78502700
O	4.59191800	-1.95942500	-1.30330600
H	5.44984400	-2.39130700	-1.37680700
O	0.09742900	-2.60314600	-0.03422800
O	3.24110300	-6.09053000	0.54060300
H	2.49400400	-6.58603000	0.89317200
C	-1.67892000	-1.10921800	-0.19668200
C	-2.35524600	-0.11003400	-0.93161000
C	-2.37420300	-1.89593800	0.73578100
C	-3.71041700	0.04275700	-0.73651800
H	-1.83381700	0.50914900	-1.64448600
C	-3.72662800	-1.70846800	0.96170600
H	-1.87286200	-2.65906600	1.31506500
C	-4.41075200	-0.74838200	0.19925600
O	-5.72985800	-0.59762900	0.33644400
H	-6.02695200	0.08565500	-0.28281600
H	0.78298100	-4.97169200	0.69670900
H	0.02706000	1.41783700	0.62050300

H	0.56999400	3.29019700	-1.73552600
H	-0.16095400	3.84747100	1.17840500
H	2.40656600	4.47218000	-0.33038800
H	1.87207200	2.46768300	1.91734700
O	4.29057400	3.62762300	1.92513100
H	5.24661700	3.59400600	2.00546600
H	4.24847400	2.77138500	0.02738900
O	-4.51637700	0.90591900	-1.39730800
C	-3.99117600	1.55657100	-2.55277300
H	-3.14478400	2.18926800	-2.28440100
H	-3.68568200	0.81172300	-3.29242400
H	-4.80407400	2.15863400	-2.94940000
O	-4.33641200	-2.51916900	1.85061400
C	-5.29548400	-1.95180400	2.75266900
H	-4.98542400	-0.94999000	3.05830000
H	-6.28164700	-1.91054700	2.29526800
H	-5.30881500	-2.61139700	3.61720700

Pt-3-*O*-glc

C	-0.78473100	3.37258500	0.85006300
C	-2.18654100	3.84850200	0.44245800
C	-3.07129300	2.63859100	0.19595700
C	-2.44682000	1.68365700	-0.80504500
C	-1.07750200	1.29998700	-0.28288900
O	-0.44177400	0.49501800	-1.25660500
C	5.56374000	-0.84699700	0.21064500
C	3.21572500	-0.98780100	-0.04430100
C	4.38953600	-1.56595300	0.40776300
C	3.16378300	0.26626500	-0.67637300
C	5.56821400	0.41689900	-0.41736400
C	1.91237900	0.75302200	-1.07792600
C	0.77107500	0.00686400	-0.87218000
C	0.86404400	-1.25657300	-0.25926700
H	6.51777600	0.92424900	-0.53919100
H	1.82097800	1.72840200	-1.54103500
O	-0.28164200	2.44233100	-0.10940500
O	-3.23263900	0.52300700	-0.94272800
H	-4.15163000	0.81889500	-0.98086300
O	-4.33280500	2.99015500	-0.31998600
H	-4.73925800	3.62316100	0.28301900
O	-2.83203200	4.57544600	1.45884200
H	-2.26062700	5.30648700	1.72521400
C	0.23578400	4.50025100	0.86602300

H	1.19345000	4.11973400	1.23469700
C	4.39124600	0.96855900	-0.85438600
O	4.28953200	2.16307000	-1.45696500
H	5.15394600	2.57216000	-1.57321900
O	2.06988600	-1.68235600	0.12636300
O	6.76063900	-1.29591200	0.59598800
H	6.70166900	-2.16085500	1.01611000
C	-0.20328300	-2.17848200	0.04272700
C	-1.41128600	-2.15532800	-0.68735000
C	0.01157100	-3.13168100	1.05368500
C	-2.37502600	-3.10294900	-0.39978500
H	-1.58100700	-1.42464900	-1.46255200
C	-0.97707200	-4.04552800	1.35905200
H	0.93177700	-3.15986200	1.62039800
C	-2.16900900	-4.03476600	0.62813700
O	-3.09485800	-4.95121300	0.94270300
H	-3.85850700	-4.85091700	0.35641700
H	4.37527200	-2.53889100	0.88173200
H	-1.16088200	0.73683100	0.66035300
H	-2.31543700	2.18897200	-1.77054800
H	-3.19083300	2.11227000	1.15573800
H	-2.10993900	4.43635600	-0.48434200
H	-0.84719700	2.90309700	1.84184200
O	-0.27457800	5.52058600	1.70681000
H	0.34028500	6.25687100	1.74478100
H	0.36601400	4.85965300	-0.16104300
O	-3.56520800	-3.25274800	-1.02246000
C	-3.77462600	-2.53370600	-2.23700100
H	-3.73431700	-1.45914700	-2.05743600
H	-3.02088900	-2.82205000	-2.97447400
H	-4.76178100	-2.82347500	-2.58635600
O	-0.78506400	-4.94382400	2.34346700
H	-1.56396100	-5.50829400	2.42670100
